# Connectivity and seasonality cause rapid taxonomic and functional trait succession within an invertebrate community after stream restoration

**DOI:** 10.1371/journal.pone.0197182

**Published:** 2018-05-24

**Authors:** Judith J. Westveer, Harm G. van der Geest, E. Emiel van Loon, Piet F. M. Verdonschot

**Affiliations:** 1 Institute for Biodiversity and Ecosystem Dynamics, University of Amsterdam, Amsterdam, The Netherlands; 2 Wageningen Environmental Research, Wageningen UR, Wageningen, The Netherlands; University of Waikato, NEW ZEALAND

## Abstract

General colonization concepts consent that a slow process of microhabitat formation and subsequent niche realization occurs during early stages after new habitat is released. Subsequently, only few species are able to colonize new habitat in the early onset of succession, while species richness increases steadily over time. Although most colonization studies have been performed in terrestrial ecosystems, running water ecosystems are equally or even more prone to colonization after disturbance due to their dynamic nature. We question how invertebrate succession patterns reconcile with general colonization concepts. With this study we provide insight into the colonization process in newly created lowland stream trajectories and answer how within-stream bio- and functional diversity develops over time. Our results show a rapid influx of species, with a wide range of functional traits, during the first season after water flow commenced. During more than two years of regular monitoring, immigration rates were highest in autumn, marking the effects of seasonality on invertebrate dispersal. Biodiversity increased while abundance peaks of species alternated between seasons. Moreover, also days since start of the experiment explains a considerable part of the variability for taxa as well as traits. However, the relative trait composition remained similar throughout the entire monitoring period and only few specific traits had significantly higher proportions during specific seasons. This indicates that first phase colonization in freshwater streams can be a very rapid process that results in a high biodiversity and a large variety of species functional characteristics from the early onset of succession, contradicting general terrestrial colonization theory.

## Introduction

Colonization is a key concept in community ecology and its study has revealed many mechanisms by which ecological communities commence and develop during ongoing succession [1–8]. Most theories describe either facilitating, tolerating or inhibiting interactions between species during succession. The main resemblance in each theory, however, is that the early successional stage is characterized by a slow sequence of microhabitat formation and houses a species poor community. Successive to the colonization of vegetation is the appearance of invertebrates, as plants provide nutrients and refuge [9, 10]. While substantial redundancy exists among theories on succession, there are still many knowledge gaps. Especially when it comes to identifying key parameters that determine the recolonization of fauna [11].

Remarkably, most colonization studies have been performed in terrestrial ecosystems, even though disturbance and subsequent succession is common in lotic ecosystems due to their dynamic nature [12, 13]. Aquatic invertebrates have traits to quickly disperse to recently released habitat [14–18], but previous studies on stream colonization have shown that succession does not merely depend on species characteristics or time itself [19]. Invertebrate succession is rather shaped by a combination of 1) the distance between the regional species pool and new habitat [20–22], 2) the presence of a suitable habitat for settling and colonization [23, 24], 3) dispersal capacity and life history traits of present species [17, 25–28], and 4) the timing of dispersal [29, 30].

Unconnected terrestrial habitat patches rely on wind-mediated dispersal, along with animal and sometimes even human vectors [31–34]. However, freshwater streams are often connected throughout a catchment and water flow is regarded as the number one facilitator of dispersal in streams [27]. As general colonization concepts seem to match terrestrial colonization more than flowing water colonization, we aim to unravel invertebrate successional patterns in newly created trajectories in temperate lowland streams.

In order to do this, we monitored three restored stream trajectories that have been connected to an existing stream channel. As previous studies have shown, the recolonizing community is shaped by the distance and composition of the surrounding species pool [21, 22] and habitat formation is affected by the inflow of allochtonous material providing a fast formation of refugia [35, 36]. This leaves us to address 1) how does taxonomic and functional trait diversity develop over time in a new stream trajectory, 2) which traits regarding dispersal capacity and life history are important during early succession, and 3) how dispersal success is affected by timing. Our expectation is a rapid increase in taxonomic as well as functional diversity right after water flow commences. New species, with different suits of functional traits, will arrive throughout the first years while the habitat is developing and more niches become available.

Metacommunity studies often neglect temporal patterns (such as ecological colonization and succession) in the interpretation of community composition, since it requires long-term and extensive monitoring. While the outcome of many metacommunity studies have led to an increased understanding of how spatial dynamics and local interactions structure communities, more empirical evidence is needed in order to fully comprehend the composition patterns in newly created habitats [37]. Therefore, we focus on temporal shifts in community composition in a restored stream. The observed temporal shift cannot be viewed independently of larger scale spatial context, but the outcome will support a mechanistic explanation for the speed at which stream communities develop after disturbance and lead to a clearer understanding of early succession patterns in lotic systems.

## Material and methods

### Study area

The area studied is located in the Leuvenumse stream (52°18’55.17”N; 5°42’33.63”E), in the province of Gelderland, the Netherlands. This is a slow-flowing, meandering lowland stream (flow velocities ranged from 8 cm/s in winter up to 46 cm/s during spates in summer), fed by both precipitation and ground water. On average, the studied stream is about 4 meters wide. The deepest part of the channel (thalweg) varies between 15–53 cm, depending on season and location. Parts of the catchment are used by agriculture. The stream itself alternates through open pasture and deciduous and coniferous woodland. The streambed substrate consists of sand and clay with patches of gravel and coarse particulate organic matter. Throughout the monitoring period, the water temperature shifted seasonally from a minimum of 3.9 °C in winter up to 17.6 °C in summer. Besides water temperature, dissolved oxygen, pH and conductivity were measured at each sampling moment (Table 1).

**Table 1:** Morphological, physical and chemical characteristics of three re-connected stream trajectories in the Leuvenumse stream, the Netherlands. These parameters were measured 18 times from September 2014 until November 2016 within each trajectory, adjacent to the streambed surface where the invertebrate samples were taken.

Parameter	minimum	maximum	average	stdev
Thalweg water depth (cm)	15	53	33	11.7
Current velocity (m/s)	0.08	0.47	0.25	0.09
pH	5.8	8.8	7.6	0.72
O_2_ (mg/L)	6.3	10.8	9.4	1.3
Conductivity (μS/cm)	209	334	290	24
Temperature (°C)	3.9	17.6	8.6	4.2

**Fig 1:**
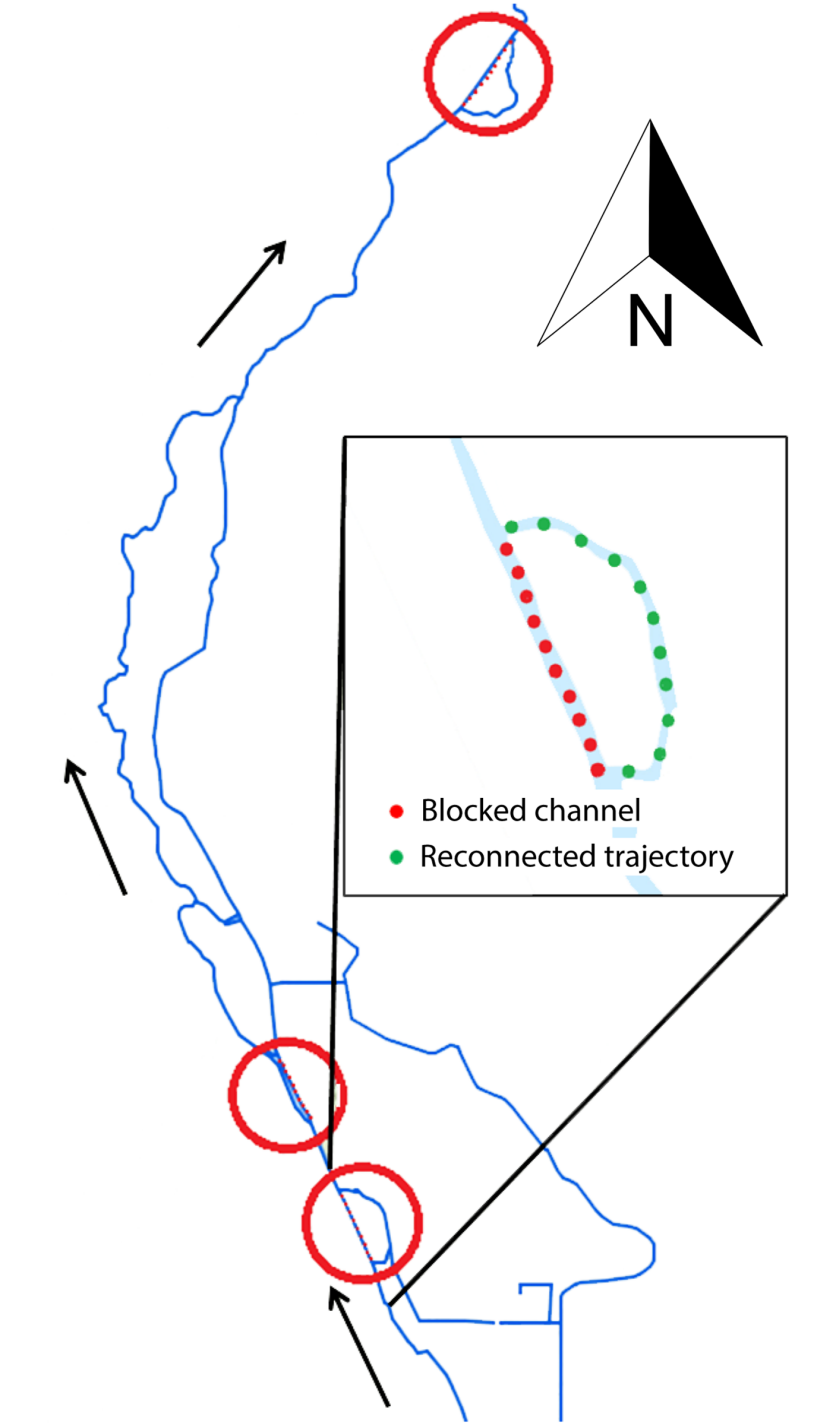
Simplified map of the Leuvenumse stream catchment with the three reconnected former stream trajectories (indicated with red circles). The arrows show the direction of the water flow. The inset shows the most upstream location; the existing channel was blocked with sand (red dots), after which the water took its natural course through the former streambed (green dots).

In the autumn of 2014, three former stream trajectories were re-connected to the existing stream by blocking parts of the main channel with sand (Fig 1). This caused all stream water to flow through the re-connected trajectories that had been dry forest floor without any pre-restoration communities up until then. All data regarding invertebrate community and morphological, physical and chemical characteristics derive from these three trajectories.

Permission to enter the national park and sample biota in the re-connected trajectories was provided by local authority NGO Natuurmonumenten with permit number 035-2014-430.

### Sample collection and processing

Within the first 6 months after water flow commenced (September—March 2014), the invertebrate community was sampled every two weeks in all three re-connected stream trajectories. After 6 months, samples were taken once per season as we assumed that community saturation slowed down the colonization rate. To sample the entire invertebrate community, three multiplate samples and two handnet samples were taken at each trajectory and each sampling moment. Hester-Dendy multiplate samplers (total sampling area is 0.10 m^2^ per sampler) were used to exclude habitat variation during colonization and placed on the stream bottom of the three new stream trajectories as soon as water started flowing (t = 0). Net samples (mesh size 250 μm, width of net is 30 cm) consisted of a multi-habitat sample composed of 1 m of mineral and 1 m of organic habitat, and were included to cover all habitat variation present at each sampling moment.

Samples were transported to the laboratory, where they were rinsed and the invertebrates picked out and stored in ethanol (70%) within 12 hours after being collected from the stream. In the laboratory, each individual was identified up to species-level if possible (Crustacea, Gastropoda, Hirudinae, Insecta: Trichoptera, Ephemeroptera, Plecoptera, Coleoptera, Simuliidae). Juveniles and some invertebrate-groups were identified up to genus-level (Bivalvia, Insecta: Chironomidae). Oligochaetes and Arachnida were excluded from the analyses due to limited identification at species-level. Care was taken to ensure that taxonomic resolution was sufficient according to Haase et al. [38]. Identification was performed with the use of a dissecting microscope (120x magnification) and a light microscope (300x magnification).

### Functional trait data

Data on functional traits was provided by freshwaterecology.info, a database that contains taxonomic and ecological information on freshwater macroinvertebrates [39], combined with trait-database EKOO [40] and Tachet’s et al. database [25]. All trait data is number coded using a 10-point system or was transformed to fit a 10-point system. We used the following six trait categories for our analyses, based on the level of importance for colonization and presence of existing knowledge for our community: feeding type, locomotion type, dispersal mode, rarity-score, number of reproductive life cycles per year and r/K-strategy. Selected functional trait data was available for respectively 94%, 92%, 68%, 65%, 41% and 23% of the 96 taxa that were found in the new trajectories during the sampling period.

### Statistical analyses

The multi-habitat and multiplate samples were pooled since the combination of taxa found by both methods represented the local community at each sampling moment. It appeared that habitat variation did not affect the data composition. No aquatic plants were present in any of the sampling sites, and streambed substrate coverage remained largely similar over time (data not included). The abiotic parameters (Table 1) did not shift over time, apart from some specific and well-known relationships between season and parameter (temperature, water depth) and were therefore not included in the analysis as an explanatory factor.

Samples of each of the three trajectories were pooled due to high community similarities (Jaccard similarity coefficient = 0.80). This enhanced the robustness of the analyses, and averaged out any local and small-scale effects.

The development of diversity and abundance over time is described with a species accumulation curve, while explicitly keeping track of new colonists. Functional diversity is investigated with the same linear model (involving season and time as predictors) on a range of trait characteristics.

The development of diversity at the species level is explored by clustering omnipresent species abundance patterns over time, with a non-metric multidimensional scaling (NMDS) ordination plot [41] and a distance based redundancy analysis [42]. To unravel community shifts in functional trait composition over time, community weighted trait means included taxa abundance per sampling moment. The explained variance in the NMDS by seasonality as well as days since the start of the experiment were evaluated. Next, the partial contribution by season and days since start were evaluated by a distance based RDA and tested by a permutation-based ANOVA at a 0.05 significance level.

To test if specific functional traits were of increasing or decreasing importance during succession, linear regressions of functional traits over time were calculated. Only taxa presence/absence data was considered in this case, multiplied with specific trait values and divided by the total number of species present per sampling moment.

All analyses were conducted in R, using functions from the package vegan for NMDS and distance-based redundancy analysis.

## Results

### Species composition

**Fig 2:**
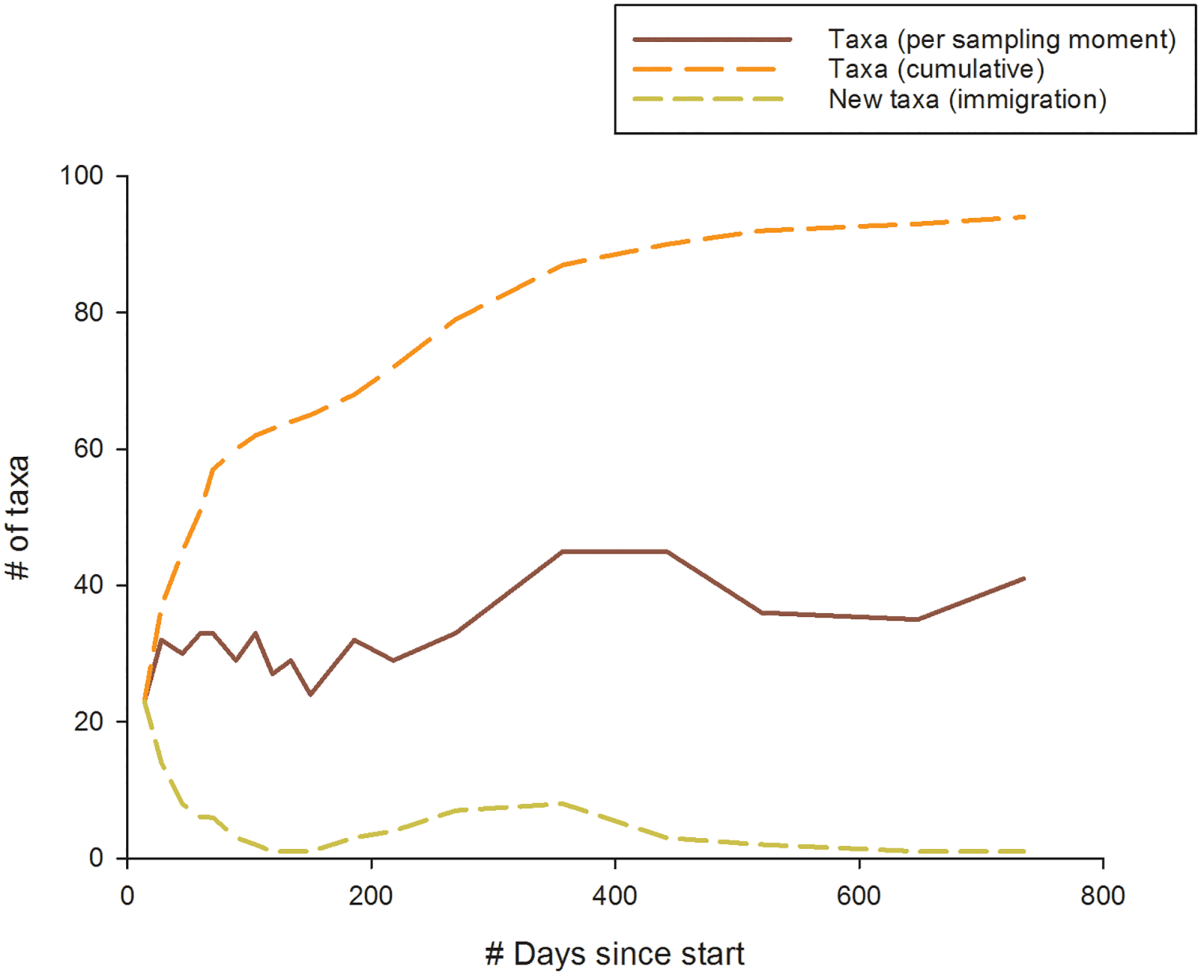
Number of taxa over time, measured 18 times from the moment water started flowing through the reconnected trajectories of the Leuvenumse stream. All three trajectories were pooled due to community similarities (Jaccard J_ij_ = 0.8). Number of taxa is plotted per sampling moment (absolute), # new taxa arriving (immigration) and # of total taxa (cumulative) over time (days since start).

In total, 96 different taxa were found in the new trajectories during the entire monitoring period of 735 days. Species diversity ranged between 23 and 45 taxa per sampling moment (Fig 2).

The colonization process started with a rapid increase in taxonomic diversity, with 23 taxa being present within the first 14 days after water flow commenced (t = 1). After 60 days (t = 4), already more than 50% of all taxa collected was present. The highest numbers of newly identified immigrating taxa is at the start in autumn (t = 1, 2 and 3) and subsequent autumn sampling moment (t = 14), exactly one year later. Towards the end of the experiment, a decreasing amount of new colonizers was observed. A linear model was used to predict differences in immigration rate based on season and time since start. A significant regression was found (F_4,13_ = 5.12, p = 0.01, R^2^ = 0.61), with more taxa arriving in autumn than winter.

**Fig 3:**
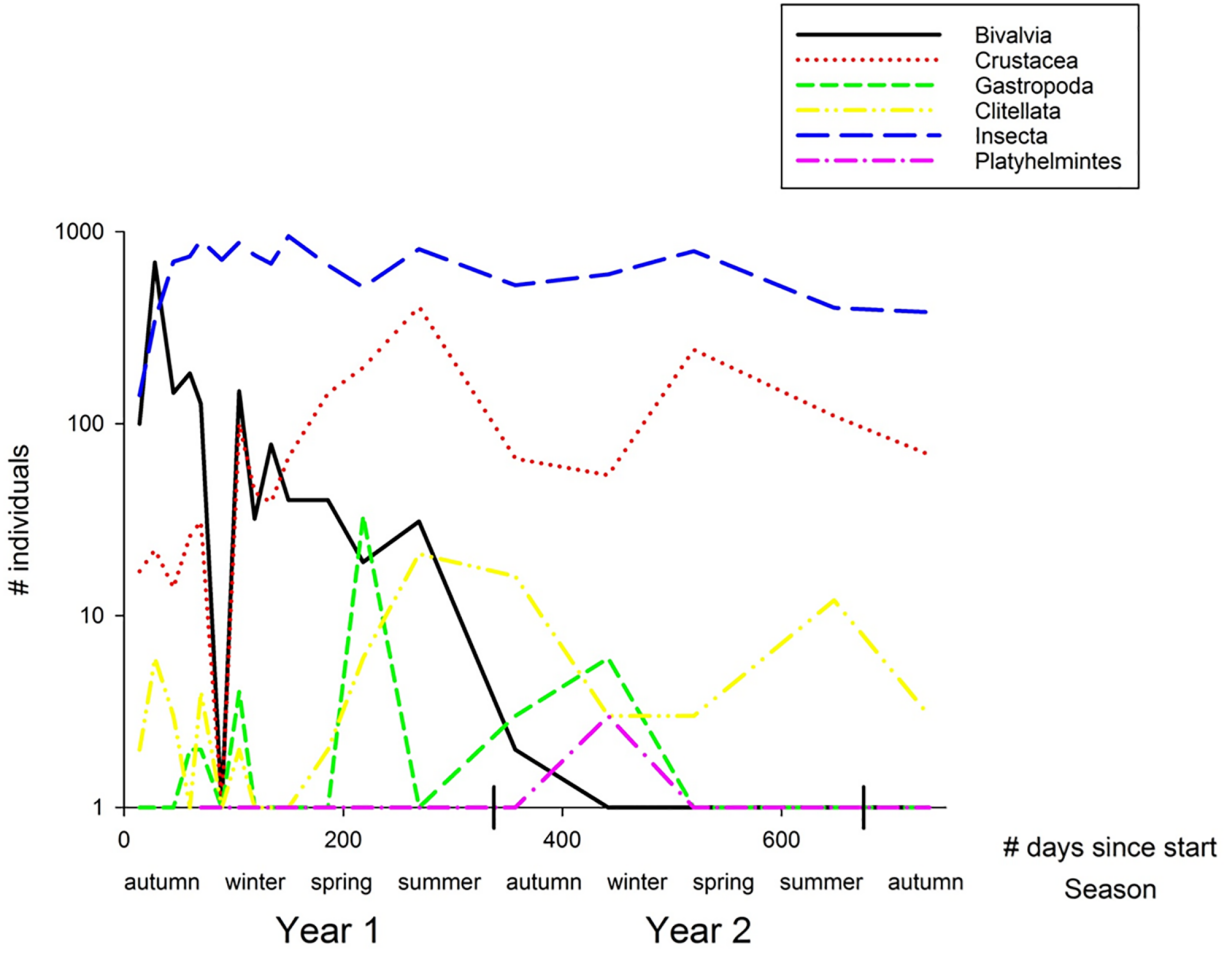
Changes in abundance (log (# individuals)) per taxonomical macroinvertebrate class over time (# days since start). Insects form the largest class of colonist from the early onset of succession, maintaining equal levels of large abundance over time.

Insects were most abundant throughout the study, ranging from 141 to 949 individuals per sample moment (average 640 ± sd 211, Fig 3). Bivalves showed abundance peaks with high numbers of individuals at the start of the colonisation (max. 642 individuals), but low abundances towards later sampling moments (average 105 ± sd 158 individual bivalves per moment). Crustaceans steadily became more abundant over time, ranging from 17 at t = 1 to 407 individuals at t = 13 (average of 44 ± sd 98 individual crustaceans per moment). The depression in bivalve and crustacean abundance at t = 6 is probably due to the occurrence of a heavy spate at the moment of monitoring.

**Fig 4:**
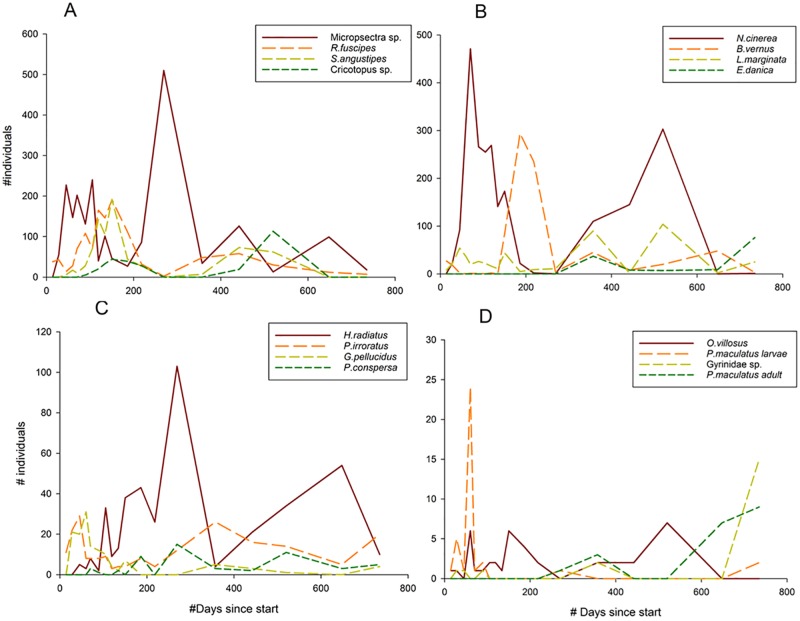
Changes in abundance over time (days since start) of four omnipresent taxa of insects, divided by order. A: Diptera, B: Ephemeroptera/Plecoptera, C: Trichoptera, D: Coleoptera.

The four most abundant orders of insects showed annual patterns with a large variation in abundance over time (Fig 4). Some taxa showed abundance peaks midwinter (Panel B. Plecoptera; *N*. *cinerea*), while other taxa were most abundant in summer (Panel C. Trichoptera; *H*. *radiatus*) or spring (Panel D. Coleoptera; *O*. *villosus*). Peaks in larval abundance are often found right after adults have oviposited their eggs. Univoltinistic patterns are found for most taxa, while some (e.g. *Micropsectra* sp.) show semivoltine or flexivoltine life cycles with more than one peak in abundance per year. Annual patterns were similar in both years.

### Functional trait composition

**Fig 5:**
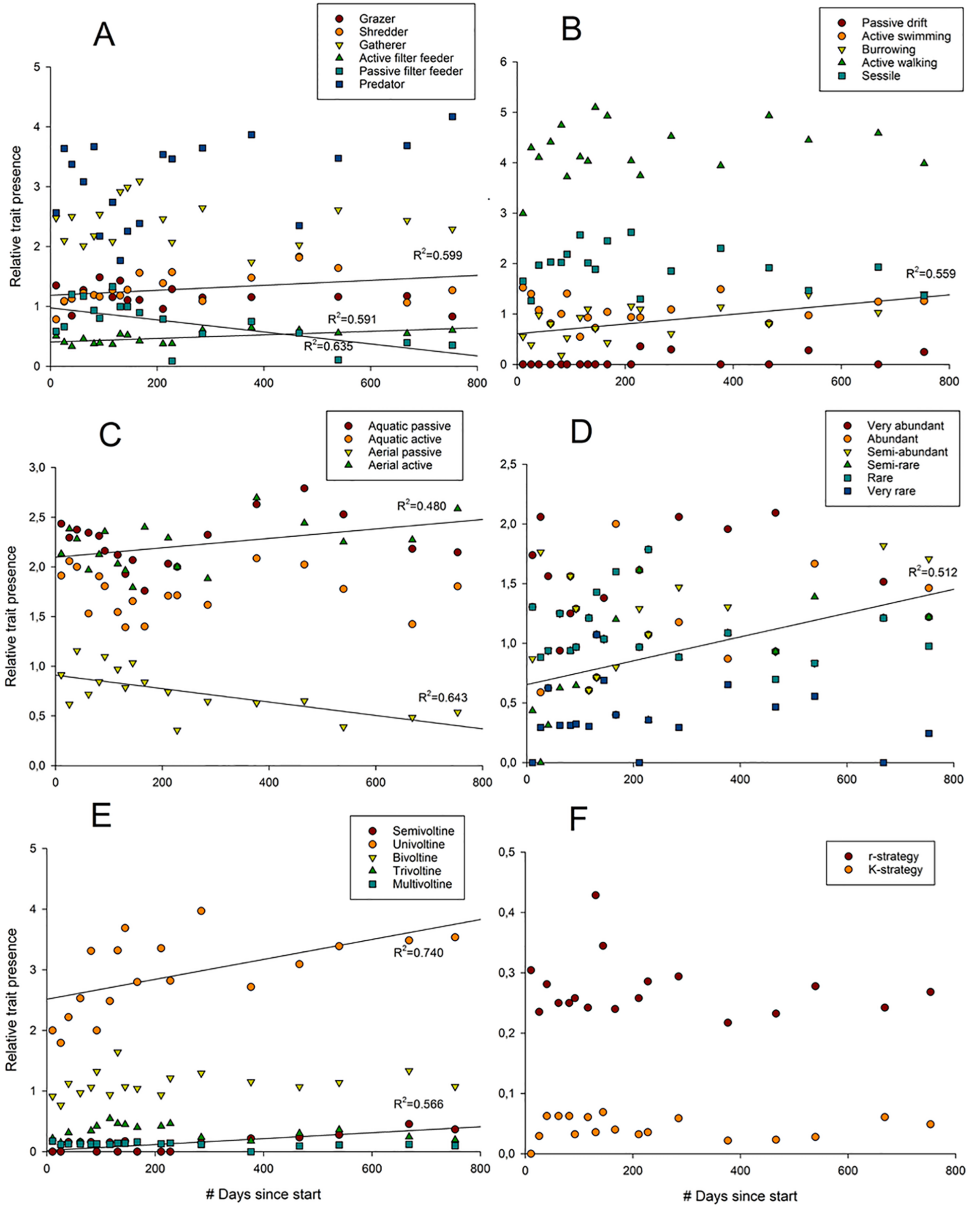
Changes in functional trait composition (relative trait presence) over time of six different trait categories. Linear regressions of functional traits over time were calculated to test if specific functional traits were of increasing or decreasing importance during succession. Only taxa presence/absence data was considered in this case, multiplied with specific trait values and divided by the total number of species present per moment. Regression lines and coefficient are only shown for significant regressions. A: Feeding group, significant regressions: shredder, active filter feeder, passive filter feeder, B: Locomotion type, sig. regr.: burrowing, C: Dispersal mode, sig. regr.: aerial passive, aerial active, D: Rarity, sig. regr: semi-rare, E: Reproductive cycle, sig. regr: semivoltine, univoltine, F: r-K selection strategy, no sig. regr.

Of the 29 traits considered in this study, divided into six different trait categories, 25 traits were present from the first sample moment onward. The overall majority of these traits (20 out of 29 traits) remained present without significant decreases and increases in relative occurrence over time (Fig 5). We do see significant increases in number of shredders, active filter feeders and burrowers (Table 2) over time. Significant increases were also found for taxa with univoltine and semivoltine reproductive cycles over time. Semi-rare taxa increased over time while rare and very rare taxa did not show significant changes. Passive filter feeders and aerial passive dispersers decreased over time.

**Table 2:** Additive linear regression model results with season (autumn, winter, spring, summer) and time since start as predictors and relative trait occurrence as dependent variable. Only significant results are shown in this table.

Trait category	Trait class	*p*	F_4,13_	R^2^
Feeding group	Shredder	0.018	4.849	0.599
Feeding group	active filter feeder	0.004	4.692	0.591
Feeding group	passive filter feeder	0.020	5.655	- 0.635
Locomotion	Burrowing	0.008	4.128	0.559
Rarity score	semi-rare	0.020	3.407	0.512
Dispersal mode	aerial passive	0.016	5.852	- 0.643
Dispersal mode	aerial active	0.014	2.996	0.480
Reproductive cycles	Semivoltine	0.000	9.265	0.740
Reproductive cycles	Univoltine	0.041	4.244	0.566

Predator taxa were most abundant (average of 34% of the total community) throughout the whole study period compared to gatherers, grazers, miners, filter feeders and shredders. Active walking taxa were most abundant (48%) throughout the whole period compared to active swimming, drifting, sessile and burrowing taxa. Taxa with a r-strategy were most abundant (87%) compared to K-strategists. Univoltinistic taxa were most abundant (62%) compared to bivoltine, trivoltine, semivoltine and multivoltine taxa. Taxa with different rarity-scores were all present in equal ratios (18%-22%), apart from the very rare taxa, which are not abundant (6%) at all times (Fig 5). Equal ratios were also present for aquatic passive and aerial active taxa, which both account for 32% of the total community.

**Fig 6:**
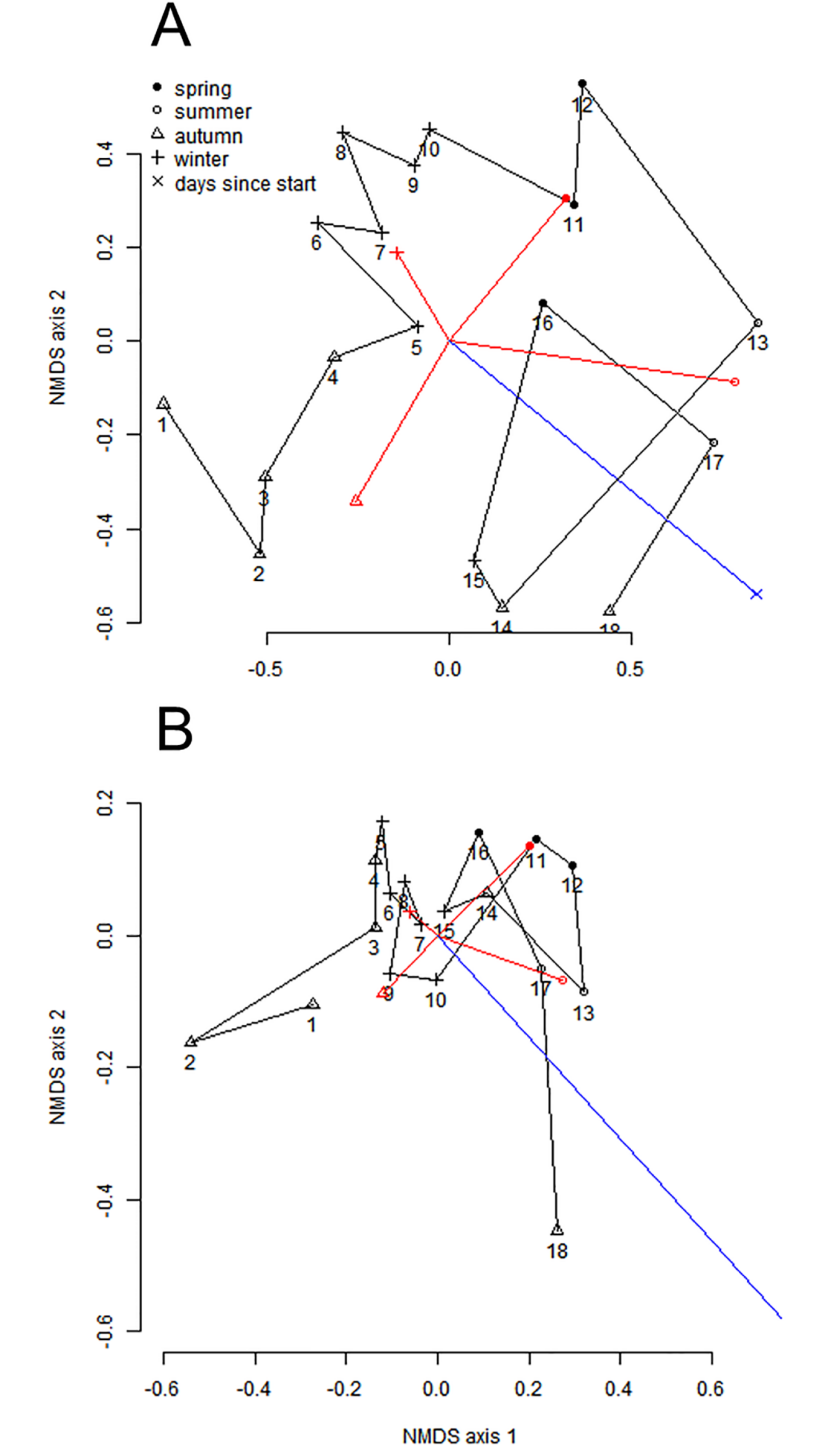
Non-metric multidimensional scaling plot (NMDS; a rank-based approach to ordination, representing the pairwise dissimilarity between all cases). For both the species (A) and the trait-based (B) analysis, the data is fitted to two axes, using the Bray-Curtis distance metric.

A NMDS of the taxa (Fig 6A) and trait composition (Fig 6B, Table 3) show that seasonality as well as days since start of experiment are important factors shaping the community composition. For the taxa-ordination, the stress value at the optimal solution is 0.159. The R-squared for the non-metric fit between observed dissimilarity and ordination distance is 0.975. Season explains the variation of the taxa in the reduced space with an r^2^ of 0.598, while days since start explains it with an r^2^ of 0.710. When analysing the contribution by season and days since start in a distance-based RDA, both factors also turn out to be significant (permutation-based p-value of 0.001 for season and 0.002 for days), with r^2^ values of 0.16, 0.40 and 0.51 for model including respectively days since start, season and days since start+season.

**Table 3:** Relative functional trait composition per season. Only significant different traits are shown. Different letters indicate significant differences between groups. Bold number indicates which season has the highest trait value.

Trait category	Trait class	Autumn	Winter	Spring	Summer
Feeding group	Grazer	a (0.79)	ab (0.88)	b **(1.74)**	ab (0.88)
Feeding group	Predator	ab (1.14)	a (0.35)	ab (0.83)	b **(1.81)**
Locomotion	passive drifting	a (0.00)	a (0.00)	a (0.00)	b **(0.04)**
Locomotion	active swimming	a (0.98)	a (0.55)	b **(3.16)**	b (2.11)
Locomotion	Sessile	ab (1.89)	a **(2.50)**	b (1.41)	ab (2.28)
Selection strategy	K-strategy	ab (0.50)	ab (0.69)	a (0.26)	b **(1.52)**
Rarity score	very common	a (0.83)	a (0.63)	b (2.30)	b **(2.92)**
Rarity score	Rare	a **(1.01)**	b (0.32)	ab (0.53)	ab (0.28)
Dispersal mode	aquatic active	a (1.93)	a (1.77)	b **(2.31)**	ab (2.04)
Reproductive cycles	Bivoltine	a (1.13)	ab (1.57)	b **(2.13)**	ab (2.08)
Reproductive cycles	Flexivoltine	a **(0.27)**	b (0.03)	ab (0.00)	ab (0.07)

For the trait-ordination, the stress value at the optimal solution is 0.11. The R-squared for the non-metric fit between observed dissimilarity and ordination distance is 0.987. Season explains the variation of the traits in the reduced space with an r^2^ of 0.418 and days since start with an r^2^ of 0.574. A distance based-RDA on the traits shows that both factors are significant (permutation-based p-value of 0.002 for season and 0.004 for days), with r^2^ values of 0.27, 0.41 and 0.58 for model including respectively days since start, season and days since start+season.

So, the differences between the observation times in terms of taxa as well as trait diversity can be represented well in a reduced (two-dimensional) space both season and days since start of the experiment explain a considerable part of the variability for taxa as well as traits.

## Discussion

This study gives insight into the shifts in community composition on a taxonomic as well as a functional trait-level during the early stages of colonization of a temperate lowland stream. Our results show that new trajectories of temperate streams are characterized by a rapid colonization by multiple species with different traits in large abundances. This observational study was performed in a highly connective system with constant supply of allochtonous organic material. These parameters could be the agent of success that caused a high rate of succession. Circumstances that are thought to increase succession rate are small distances to the regional source pool [22, 34], species with active dispersal mechanisms [43, 44], specific seasonal effects [30] and suitable new habitat. We will discuss how these four elements have played a role in our study and how they affect the process of succession in temperate lowland streams.

### Distance and dispersal mechanisms

Parkyn and Smith [45] consider dispersal constraints low if a new habitat has an upstream supply of invertebrates and additional aerial recolonists are no further than 2 km away. Under such conditions the new habitat could resemble a reference community within 10–50 years after disturbance. Sundermann et al. [22] and Winking et al. [17] consider dispersal constraints high when there is no direct upstream community and recolonists are more than 5 km away. Those communities are very unlikely to resemble a reference community ever again. Recent studies have shown that succession can be a slow process if the connectivity between streams is poor [45, 46, 47].

In our case, the re-connected trajectories were directly, and imperatively, connected to the upstream and downstream existing stream channel and therefore dispersal constraints were considered low. Furthermore, hydrological connectivity and riparian vegetation was present at all times, which enabled rapid colonization of aerial and aquatic dispersers from the start. Even though there is still a debate on which pathway makes up the majority of dispersal, it has been found that short-distance dispersal mainly occurs due to aquatic drift [22, 45, 48] while long-distance dispersal by adult insect flight is often the primary mechanism for recolonization of restored trajectories in separate catchments [49, 50]. However, many species are incapable of actively dispersing themselves and rely on animal vectors, wind, or water flow to provide passive transport between sites [19]. In terrestrial ecosystems, wind-mediated dispersal is the prime mechanism that enables early stage succession [51]. In running waters, flow facilitates colonization from the first moment on [52, 53] and connectivity to source populations will further speed up arrival.

All four dispersal mechanisms (aquatic/aerial active/passive) were present among taxa from the onset of succession. Aerial passive dispersers decreased over time, all other dispersal mechanisms remained present at equal ratios over time. This finding contradicts recent studies where aerial active generalists were found to colonize most rapidly and weakly dispersing generalists immigrated much later [17, 28]. This contradiction and our observation leads us to hypothesize that categorizing species by dispersal mechanisms alone does not give sufficient information on actual dispersal rate.

A clearer view of how the community developed during the process of succession is obtained by looking at the functional trait composition. We found that while some traits were dominant over others, overall trait composition did not change over time. For terrestrial systems, new habitat is colonized by species that have rapid life cycles and are r-strategists. In later stages, species with competitive traits, such as predatory behavior, are able to settle in the new habitat. Analogous, Gore [20] showed that collector-gatherers and collector-filterers were the initial colonizers of stream habitat instead of predators. Surprisingly, the traits found to be dominant in this study contradict the colonizer-competitor trade-off concept [7, 54, 55] and the findings of Gore [20]. From the onset of succession, predatory species were present in the new habitat. The diversity of predators was even higher than the diversity of filter feeders. In accordance with other studies [20, 28, 48, 56] our study shows that the trait composition of the newly arriving species community probably depends on the regional species pool in the proximity, rather than develop according to previously theorized sequence of functional traits.

### Succession and seasonal effects

Our results indicate that there is a temporal trend for the duration of the project (viz the results by distance-based RDA), which might be successional. On top of that there are clear seasonal effects (also shown in distance-based RDA). The results show that most species either immigrate in the first season or exactly one year later in the same season. Both immigration events took place in autumn. This indicates the importance of seasonality during the colonization process in temperate running waters. Seasonality can effect dispersal processes directly, in terms of optimal hydrological connectivity [57] and species availability due to life-cycle timing [58–60], and indirectly, in terms of increases and decreases of specific environmental parameters that trigger organisms to disperse [30]. Peak flows after heavy rainfall are common in Dutch lowland streams during the end of summer and autumn. The sudden increase in current velocity can cause species to become dislodged from the substrate and enter a state of drift [61]. Additionally, an increase in the volume of water due to melting or rainfall can expand potential habitat surface and increase hydrological connectivity. This improves instream dispersal and colonization patterns [62]. In the late summer and early autumn temperate lowland streams are populated by many juvenile and first larval stages specimens of many species [63–65]. These young animals are often susceptible to drift, either passively or actively as dispersal trait, and prone to transportation downstream where they can settle.

Throughout the year, also some new species immigrate into the denuded habitat. This can be explained by both ongoing habitat development and the ability of species to inhabit these new habitats due to their functional traits [66, 67]. Interestingly, species from different insect orders show annual peaks in abundance, but alternate in the timing of these peaks. By partitioning resources and interspecific competition, more species are able to coexist while total abundance levels are stable and succession continues [12].

The result that species and trait composition is correlated to seasonality indicates that seasonal life history characteristics provide the opportunity to colonize, survive and reproduce and not just time since existence or dispersal capacity alone. This finding is supported by previous studies on stream restoration effects [56], and can be explained by the different life history strategies needed to exploit (a)biotic resources [67, 68]. Surprisingly, little attention has been given to seasonal patterns in macroinvertebrate colonization in temperate streams. In our study, the combination of strong hydrological connectivity, high availability of juveniles, and availability of habitats and resources of re-connected trajectories set the requisites for enhanced autumn colonization.

### Habitat suitability

Once the macroinvertebrates arrive at the newly restored habitat, they have to be able to settle and reproduce before recruitment can be considered successful. However, the presence of favorable in-stream and riparian habitat alone is not enough for community recovery [69]. Distances between leaf patches and sizes of these organic refuges have a profound effect on species survival [70, 71]. Species might not be able to recolonize the stream before multiple new microhabitats have established in the stream [35, 36]. The rapid increase in biodiversity and functional diversity right after water flow commenced indicates that microhabitat formation was not a limiting factor for the first species to arrive. High hydrological connectivity between the upstream source pool of organic matter and the reconnected trajectory together with the input of allochtonous material from the autumn-shed leaves along this temperate lowland stream could have provided a fast formation of suited habitats.

To conclude, this study shows that temperate lowland stream colonization is marked by a rapid increase in species richness and abundance right after water flow commences, when hydrological connectivity is not a constraint. The community is heterogeneous in terms of functional trait diversity from the early onset, with no clear sequence in colonizer to competitor trait characteristics. The immigration rate of new species is affected by seasonality. To strengthen the interpretation of these observations, data from nearby established streams help to standardize the community composition by season and determine at what time point the assembled communities of new streams become statistically indistinguishable from established streams. This could unravel the proportion of both spatial and temporal effects on successional patterns. Nonetheless, the observed patterns show that colonization and subsequent succession rate can be high in restored stream trajectories, yet effects of the regional species pool might be a limiting factor in community restoration and worth to explore in future studies.
